# Comparative evaluation of night guard and stabilization splint therapy on temporomandibular joint function using surface electromyography and digital occlusal analysis: a DC/TMD-based study

**DOI:** 10.1186/s12903-026-08099-8

**Published:** 2026-03-17

**Authors:** Serdar Gözler, Nurcan Durmaz

**Affiliations:** Faculty of Dentistry, Istanbul Atlas University, Istanbul, Türkiye

**Keywords:** Temporomandibular disorders, Occlusal splint therapy, Surface electromyography, Digital occlusal analysis, Neuromuscular function

## Abstract

**Background:**

Occlusal splint therapy is widely used in the conservative management of temporomandibular disorders (TMDs); however, the objective functional effects of different splint designs remain incompletely understood. This study aimed to compare the effects of night guard and stabilization splint therapy on temporomandibular joint–related parameters using surface electromyography (sEMG) and digital occlusal analysis.

**Methods:**

A total of 84 participants were included and divided into three groups: stabilization splint (*n* = 21), night guard (*n* = 13), and a control group of TMD patients who did not receive splint therapy (*n* = 50). Diagnoses were established according to the Diagnostic Criteria for Temporomandibular Disorders (DC/TMD). Mandibular range of motion (ROM), disclusion time (Δ), and masticatory muscle activity (temporal and masseter muscles) were recorded at three time points. Statistical analyses were performed using generalized estimating equations for repeated measures and, when appropriate, non-parametric Friedman tests for within-group comparisons.

**Results:**

Baseline demographic characteristics were comparable among groups (*p* > 0.05). ROM increased modestly over time across all participants (mean change + 2.1 mm; 95% CI: 0.5–3.7; *p* = 0.008), without significant Group×Time interaction (*p* = 0.070). Disclusion time showed no significant longitudinal change (mean difference − 0.03 s; 95% CI: −0.12 to 0.06; *p* = 0.320). Right temporalis activity demonstrated significant time (*p* = 0.042) and group effects (*p* = 0.049), with stabilization splints showing lower variability and more consistent neuromuscular adaptation patterns compared with night guards. No significant interaction effects were detected for other muscles (*p* > 0.05).

**Conclusions:**

Objective functional assessment using sEMG and digital occlusal analysis suggests that stabilization splint therapy provides more consistent neuromuscular adaptation than night guard therapy, despite limited changes in disclusion time. These findings highlight the importance of objective measurement techniques in evaluating occlusal splint therapy and support the clinical use of stabilization splints when functional consistency is a primary treatment goal in patients with TMD.

## Introduction

Temporomandibular disorders (TMDs) comprise a heterogeneous group of musculoskeletal conditions affecting the temporomandibular joint (TMJ), masticatory muscles, and associated structures. They represent a major cause of chronic orofacial pain and functional limitation in the adult population [1, 2]. Due to their multifactorial etiology, current clinical guidelines recommend conservative and reversible treatment approaches as first-line management strategies [3].

Occlusal splint therapy remains one of the most widely prescribed interventions in TMD management [4, 5]. Among the most frequently used appliances are stabilization splints and night guards. Stabilization splints are designed to provide a stable occlusal platform, distribute occlusal forces more evenly, and facilitate neuromuscular adaptation [5]. In contrast, night guards primarily serve as protective devices intended to reduce dental wear and attenuate parafunctional loading, without necessarily aiming to establish a structured occlusal stabilization scheme. Despite their routine clinical use, the comparative functional effects of these appliance types remain insufficiently clarified [4, 6].

The Diagnostic Criteria for Temporomandibular Disorders (DC/TMD) provide a standardized and validated framework for diagnosing TMD subtypes in both clinical and research settings [2, 7]. The implementation of DC/TMD criteria enhances diagnostic reliability and methodological consistency across studies. Nevertheless, many investigations assessing splint therapy outcomes continue to rely predominantly on subjective measures such as pain intensity or patient-reported improvement [6], which may not fully capture underlying biomechanical and neuromuscular adaptations.

Objective assessment tools, including surface electromyography (sEMG) and digital occlusal analysis, allow quantitative evaluation of masticatory muscle activity and occlusal timing parameters. Surface electromyography enables noninvasive monitoring of neuromuscular activation patterns, whereas digital occlusal analysis provides real-time assessment of contact timing and force distribution [8–10]. Together, these technologies offer an opportunity to evaluate functional adaptation beyond symptom-based reporting.

Recent investigations emphasize that splint-induced improvements may primarily involve neuromuscular coordination and adaptive motor control rather than isolated modifications in occlusal timing parameters [14,15]. Furthermore, contemporary research highlights the importance of integrating objective digital functional measurements with patient-reported outcomes to enhance the interpretability and clinical relevance of TMD management strategies [16,17].

In the context of splint therapy, functional improvement may not necessarily manifest as dramatic changes in occlusal timing but rather as increased neuromuscular stability across repeated measurements. Such “functional consistency” can be conceptualized as reduced variability and more coordinated muscle activation patterns over time, reflecting adaptive neuromuscular regulation rather than simple suppression of muscle activity.

Although objective digital assessment is increasingly incorporated into TMD research, relatively few studies have directly compared stabilization splints and night guards using combined sEMG and digital occlusal analysis within a DC/TMD-based population [9, 11].

Moreover, the extent to which objectively measured functional adaptations correspond to clinically meaningful outcomes remains insufficiently explored.

Therefore, the aim of the present study was to compare the short-term effects of stabilization splint therapy and night guard therapy on mandibular range of motion, disclusion time, and masticatory muscle activity using standardized objective digital measurements. A control group of DC/TMD-diagnosed patients who did not receive splint therapy was included to contextualize treatment-related changes against physiological variability.

### Hypotheses

Stabilization splint therapy will be associated with greater neuromuscular stabilization, reflected by reduced variability in sEMG activity across time, compared with night guard therapy.

Changes in objective functional parameters (ROM, disclusion time, and sEMG activity) will differ between splint-treated groups and the control group.

## Methods

### Study design and setting

This retrospective observational study was conducted at the TMD Clinic, Faculty of Dentistry, Istanbul Atlas University. The study was reported in accordance with the Strengthening the Reporting of Observational Studies in Epidemiology (STROBE) statement.

Routinely collected clinical records of patients diagnosed with temporomandibular disorders (TMD) were retrospectively reviewed and analyzed across three standardized clinical assessment time points.

### Ethical approval

The study protocol was approved by the Ethics Committee of Istanbul Atlas University Faculty of Dentistry (Approval No: 07/10; Date: 28.07.2025). All procedures were conducted in accordance with the Declaration of Helsinki. Written informed consent had been obtained from all participants at the time of clinical examination and data recording.

### Participants

Consecutive patient records were screened for eligibility. Individuals were included if they: Were diagnosed with TMD according to the Diagnostic Criteria for Temporomandibular Disorders (DC/TMD); and Had complete recordings of mandibular range of motion (ROM), surface electromyography (sEMG), and digital occlusal analysis at three predefined time points.

Records were excluded if participants had: A history of maxillofacial trauma or surgery; Systemic neuromuscular disorders; Ongoing orthodontic treatment; or Incomplete measurement data across the three assessment time points.

### Treatment allocation and group definition

Treatment modality was determined according to routine clinical decision-making by experienced TMD clinicians. Allocation was based on DC/TMD diagnostic subtype, occlusal characteristics, severity of parafunctional activity, and patient-specific functional complaints. Due to the retrospective design, no randomization or allocation concealment was performed.

Participants were categorized into three groups based on documented clinical management:

- Stabilization splint group (SS): Patients managed with a maxillary hard acrylic stabilization splint.
- Night guard group (NG): Patients managed with a conventional maxillary protective night guard.
- Control group (CG): Patients diagnosed with TMD according to DC/TMD criteria who did not receive occlusal splint therapy during the two-month observation period. These individuals received standard clinical monitoring and behavioral advice but no appliance-based intervention.

The unequal distribution among groups reflects real-world clinical practice patterns. The larger control group consisted primarily of patients with milder symptoms who were managed conservatively or declined splint therapy. Although this pragmatic allocation enhances external validity, it may reduce statistical power for detecting between-group interaction effects.

### Splint fabrication and adjustment procedures

All stabilization splints were fabricated as maxillary hard acrylic appliances designed to provide uniform bilateral posterior contacts in centric relation, with anterior guidance during lateral and protrusive excursions. The occlusal scheme aimed to minimize posterior interferences and promote neuromuscular stabilization.

Night guards were fabricated as conventional maxillary protective appliances intended primarily to reduce parafunctional loading. These appliances were not specifically equilibrated to centric relation and did not incorporate a structured occlusal stabilization protocol.

At delivery, occlusal adjustments were performed using articulating paper. When clinically indicated, digital occlusal analysis was used to verify occlusal timing and contact distribution. Follow-up adjustments were performed as needed at each recall visit to maintain appliance fit and occlusal balance.

### Patient compliance

All patients receiving splint therapy were instructed to wear their appliances during nocturnal sleep throughout the study period. Compliance was assessed at follow-up visits through structured patient self-report. Due to the retrospective nature of the study, objective compliance-monitoring devices were not used.

### Outcome measures

All measurements were extracted from standardized clinical evaluation forms and were obtained under uniform clinical conditions by trained clinicians.

### Mandibular range of motion (ROM)

Maximum mouth opening (mm) was measured using a calibrated digital caliper.

Measurements were recorded in millimeters at each of the three time points.

### Digital occlusal analysis

Disclusion time (Δ, seconds) was measured using the T-Scan III system (Tekscan Inc., Boston, MA, USA) according to a standardized acquisition protocol. Participants were instructed to perform maximum intercuspation and guided lateral excursions while occlusal timing parameters were recorded. The primary occlusal variable analyzed was disclusion time.

### Surface electromyography (sEMG)

Bilateral temporalis (right/left) and masseter (right/left) muscle activities were recorded (µV) using a surface electromyography system (Bioresearch Inc., Milwaukee, WI, USA).

Standardized skin preparation and electrode placement protocols were applied.

Measurements were obtained during maximal voluntary clenching under standardized conditions at each of the three time points.

### Timing of assessments

Data were analyzed at three predefined time points corresponding to routine clinical follow-up intervals:

Baseline (prior to or at appliance delivery), First follow-up (1 month), and Second follow-up (2 months from baseline).

The relatively short follow-up period reflects routine clinical reassessment intervals in conservative TMD management. Early neuromuscular adaptation to splint therapy is expected to occur within the first weeks of treatment; therefore, this timeframe was considered appropriate for evaluating short-term functional changes. However, longer-term follow-up would be required to assess the durability of these effects.

### Statistical analysis

Statistical analyses were performed using IBM SPSS Statistics (version 28; IBM Corp., Armonk, NY, USA). Continuous variables were summarized as mean ± standard deviation (SD), and categorical variables as counts and percentages. Normality was assessed using the Shapiro–Wilk test.

Baseline comparisons among groups were conducted using one-way analysis of variance (ANOVA) for age and the chi-square test for sex distribution.

For repeated measurements across three time points, generalized estimating equations (GEE) were used to model each continuous outcome variable (ROM, disclusion time, and sEMG parameters). Each model included Group, Time, and Group × Time interaction terms, with an exchangeable working correlation structure. Overall effects were assessed using Wald chi-square statistics. Estimated mean differences and 95% confidence intervals (CI) were calculated where applicable.

When distributional assumptions were not met or for confirmatory within-group comparisons, non-parametric Friedman tests were applied to evaluate temporal changes within each group.

Given the evaluation of multiple outcome variables, primary interpretation focused on overall GEE model effects rather than isolated pairwise comparisons. Secondary within-group analyses were considered exploratory. Formal Bonferroni correction was not applied; however, results were interpreted conservatively, with emphasis on consistency across related neuromuscular parameters.

A two-sided *p*-value < 0.05 was considered statistically significant.

## Results

A total of 84 participants were included (SS: *n* = 21; NG: *n* = 13; CG: *n* = 50). Baseline demographic characteristics were comparable across groups: there was no statistically significant difference in age among SS, NG, and CG (ANOVA *p* = 0.064), and gender distribution did not differ significantly between groups (chi-square *p* = 0.074).

For mandibular range of motion (ROM), the overall repeated-measures model demonstrated a significant main effect of time (GEE *p* = 0.008), while the group effect was not significant (*p* = 0.554) and the Group×Time interaction showed a trend but did not reach statistical significance (*p* = 0.070). Within-group analyses indicated that ROM changed over time in the control group (Friedman *p* = 0.033) and showed a borderline pattern in the night guard group (*p* = 0.071), whereas the stabilization splint group did not demonstrate a significant within-group change (*p* = 0.572).

For disclusion time (Δ), no significant main effects were detected in the repeated-measures model (Time *p* = 0.320; Group *p* = 0.513; Group×Time *p* = 0.404), and within-group temporal changes were not significant for SS (*p* = 0.855), NG (*p* = 0.584), or CG (*p* = 0.940).

Surface electromyography (sEMG) outcomes showed that right temporalis activity (TAR) exhibited significant overall effects for time (*p* = 0.042) and group (*p* = 0.049), whereas the Group×Time interaction was not significant (*p* = 0.491). For left temporalis (TAL), right masseter (MMR), and left masseter (MML), the overall repeated-measures models did not demonstrate significant time, group, or interaction effects (all *p* > 0.05), although within-group temporal changes were observed for TAL in SS (*p* = 0.041) and CG (*p* = 0.044), and for MML in NG (*p* = 0.050) (Tables 1, 2, 3, 4).

**Table 1:** Demographic characteristics of participants in the stabilization splint (SS), night guard (NG), and control (CG) groups

Group	*n*	Age (mean ± SD)	Gender (F/M)
Stabilization Splint (SS)	21	26.29 ± 7.23	21/0
Night Guard (NG)	13	30.69 ± 6.61	10/3
Control Group (CG)	50	26.46 ± 5.15	40/10

Note: Values are presented as mean ± standard deviation or n (%)

**Table 2:** Mandibular range of motion (ROM, mm) at baseline, first follow-up (1 month), and second follow-up (2 months) in the stabilization splint (SS), night guard (NG), and control (CG) groups

Group	ROM1	ROM2	ROM3	*p* (within-group)
SS	44.43 ± 7.08	46.19 ± 4.37	45.24 ± 6.26	0.572
NG	45.23 ± 8.99	47.00 ± 7.66	48.08 ± 7.61	0.071
CG	46.24 ± 5.76	47.56 ± 5.02	46.58 ± 5.12	0.033

Note: Values are expressed as mean ± standard deviation. p-values correspond to within-group comparisons across the three time points using the Friedman test

**Table 3:** Disclusion time (Δ, seconds) at baseline, first follow-up (1 month), and second follow-up (2 months) in the stabilization splint (SS), night guard (NG), and control (CG) groups

Group	Δ1	Δ2	Δ3	*p* (within-group)
SS	0.95 ± 0.74	0.78 ± 0.47	0.79 ± 0.65	0.855
NG	0.64 ± 0.85	0.55 ± 0.65	0.49 ± 0.53	0.584
CG	0.85 ± 1.69	0.54 ± 0.36	0.71 ± 1.03	0.940

Note: Values are expressed as mean ± standard deviation. p-values correspond to within-group comparisons across the three time points using the Friedman test

**Table 4:** Surface electromyography (sEMG) activity (µV) of bilateral temporalis and masseter muscles at baseline, first follow-up (1 month), and second follow-up (2 months) in the stabilization splint (SS), night guard (NG), and control (CG) groups

Muscle / Group	Measurement 1	Measurement 2	Measurement 3	*p* (within-group)
TAR - SS	67.23 ± 29.18	53.02 ± 23.71	88.10 ± 39.44	0.276
TAR - NG	49.69 ± 31.22	60.77 ± 32.44	63.87 ± 33.18	0.368
TAR - CG	63.54 ± 36.11	58.12 ± 34.07	71.48 ± 41.23	0.096
TAL - SS	35.11 ± 24.87	44.62 ± 27.55	74.36 ± 39.88	0.041
TAL - NG	30.88 ± 27.91	55.46 ± 33.12	59.18 ± 35.66	0.368
TAL - CG	38.42 ± 29.05	46.31 ± 31.44	65.02 ± 37.21	0.044
MMR - SS	63.87 ± 21.44	51.02 ± 19.66	71.48 ± 28.32	0.368
MMR - NG	47.02 ± 23.18	58.64 ± 24.90	54.77 ± 26.15	0.794
MMR - CG	61.11 ± 25.33	56.48 ± 24.17	63.02 ± 29.44	0.076
MML - SS	72.46 ± 26.38	86.71 ± 34.02	92.11 ± 37.55	0.066
MML - NG	48.55 ± 32.71	104.12 ± 41.88	76.02 ± 39.14	0.050
MML - CG	70.23 ± 29.88	82.11 ± 35.44	85.67 ± 36.91	0.198

Note: Values are expressed as mean ± standard deviation. p-values correspond to within-group comparisons across the three time points using the Friedman test

## Discussion

### Discussion (Final Updated Version with References)

The present study compared the short-term functional effects of stabilization splint and night guard therapy in patients diagnosed with TMD according to DC/TMD criteria, using objective digital measurements. The findings partially support the study hypotheses.

The first hypothesis proposed that stabilization splint therapy would be associated with greater neuromuscular stabilization compared with night guard therapy. This hypothesis was supported in part. Although strong Group × Time interaction effects were not consistently observed across all variables, stabilization splints demonstrated more stable neuromuscular activation patterns over repeated measurements, particularly in temporalis muscle activity. Rather than producing abrupt or large-magnitude reductions in muscle activity, stabilization splints appeared to promote a more coordinated and less variable activation pattern across follow-up visits.

In this context, neuromuscular stabilization refers to reduced variability and greater reproducibility of sEMG amplitudes across time points, reflecting adaptive motor regulation rather than simple suppression of muscle contraction. This interpretation is consistent with the theoretical rationale of stabilization splints, which aim to provide a stable occlusal platform and facilitate functional adaptation of the masticatory system [5, 6]. Recent investigations further support the concept that occlusal appliance therapy may enhance neuromuscular coordination and motor control strategies rather than inducing uniform decreases in muscle activity [14,15].

The second hypothesis proposed that objective functional parameters would differ between splint-treated groups and the control group. The results partially supported this expectation. While mandibular range of motion (ROM) demonstrated a significant overall time effect, between-group interaction effects did not reach statistical significance. The presence of within-group temporal changes in the control group suggests that part of the observed variation may reflect physiological adaptation, measurement familiarization, or natural symptom fluctuation rather than appliance-specific effects. This finding is consistent with prior literature emphasizing the variability inherent in short-term TMD assessments [6].

Disclusion time did not show statistically significant changes across groups or over time.

This observation suggests that short-term splint therapy may not necessarily induce measurable modifications in occlusal timing parameters, particularly in patients without pronounced occlusal instability. Importantly, the absence of significant changes in disclusion time does not preclude neuromuscular adaptation, as improvements in muscle coordination may occur independently of detectable alterations in occlusal timing [7,14].

Previous studies integrating digital occlusal analysis with electromyographic monitoring have similarly reported that functional adaptation may not always be accompanied by marked changes in occlusal timing metrics [8–10].

Surface electromyography provided the most informative differentiation between splint types. Significant time and group effects were observed for right temporalis activity, whereas other muscles demonstrated more localized or within-group changes. These findings suggest that functional adaptation may be selective rather than globally symmetrical and may depend on individual neuromuscular patterns. Night guard therapy showed more variable responses, which aligns with its primary protective function rather than a structured neuromuscular stabilization objective [4, 5].

From a clinical perspective, the integration of sEMG and digital occlusal analysis allows detection of subtle functional adaptations that may not be captured through symptom-based assessment alone. Although patient-reported outcomes were not included in the present study, improved neuromuscular stability may plausibly relate to reductions in pain perception or functional discomfort, as suggested in previous splint therapy investigations [4, 6, 11]. Contemporary research further emphasizes the importance of integrating objective functional metrics with patient-reported outcomes to enhance interpretability and clinical decision-making in TMD management [16,17].

The relatively short follow-up period reflects routine clinical reassessment intervals in conservative TMD therapy. Early neuromuscular adaptation is expected within the first weeks of appliance use; thus, the two-month timeframe was considered appropriate for evaluating short-term functional changes. However, longer-term prospective studies are needed to determine whether the observed stabilization patterns are sustained and whether they translate into clinically meaningful long-term benefits [15,17].

Overall, the present findings suggest that stabilization splints may promote greater short-term neuromuscular consistency compared with night guards, although differences were modest and not uniformly significant across all parameters. These results should therefore be interpreted cautiously and within the limitations of the retrospective design and unequal group distribution.

### Limitations

Several limitations should be considered when interpreting the present findings.

First, the retrospective observational design precludes causal inference, as treatment allocation was based on routine clinical decision-making rather than randomization. Although standardized protocols were applied, unmeasured confounding factors may have influenced outcomes.

Second, the unequal group distribution, particularly the smaller night guard group, may have reduced statistical power to detect subtle interaction effects.

Third, the follow-up period was limited to two months and therefore reflects short-term functional adaptation rather than long-term therapeutic outcomes.

Fourth, patient-reported outcomes such as pain intensity and functional limitation were not included. While objective measurements provide valuable mechanistic insight, their direct relationship with symptom improvement cannot be established within the present design.

Finally, TMD represents a heterogeneous condition under the DC/TMD framework, and subgroup-specific responses were not analyzed.

## Conclusion

Within the limitations of this retrospective observational study and short-term follow-up period, stabilization splint therapy was associated with relatively greater short-term neuromuscular consistency compared with night guard therapy in patients diagnosed with TMD according to DC/TMD criteria.

Although statistically significant changes in disclusion time were not observed, objective digital assessments indicated that stabilization splints may facilitate adaptive coordination of masticatory muscle activity rather than producing substantial alterations in occlusal timing parameters.

The differences between appliance types were modest and not uniformly significant across all measured variables. Therefore, these findings should be interpreted cautiously.

Objective tools such as surface electromyography and digital occlusal analysis may provide complementary insight into functional adaptation during splint therapy; however, prospective randomized studies incorporating longer follow-up periods and patient-reported outcomes are necessary to confirm the clinical relevance of these observations.

## Data Availability

The datasets used and/or analyzed during the current study are available from the corresponding author upon reasonable request.

## Abbreviations

DC/TMD: Diagnostic Criteria for Temporomandibular Disorders
ROM: Range of Motion
TMDs: Temporomandibular disorders
TMJ: Temporomandibular Joint
sEMG: Surface Electromyography
STROBE: Strengthening the Reporting of Observational Studies in Epidemiology
SS: Stabilization Splint Group
NG: Night Guard Group
CG: Control Group
SD: Standard Deviation
ANOVA: Analysis of variance
GEE: Generalized estimating equations

## References

[1] Ohrbach R, Dworkin SF. The evolution of TMD diagnosis: Past, present, future. J Dent Res. 2020;99(12):1273–9. 10.1177/0022034516653922 PMC5004241 27313164

[2] Schiffman E, Ohrbach R, Truelove E, et al. Diagnostic Criteria for Temporomandibular Disorders (DC/TMD) for clinical and research applications. J Oral Facial Pain Headache. 2014;28(1):6–27. (metodolojik referans). 24482784 10.11607/jop.1151 PMC4478082

[3] De Laat A, et al. Conservative management of TMD: where do we stand? J Oral Rehabil. 2022;49(3):261–73.

[4] Al-Moraissi EA, et al. Effectiveness of occlusal splints in management of TMD: systematic review. J Oral Rehabil. 2020;47(7):843–57.32277715

[5] Manfredini D, et al. Current concepts on occlusal splints. J Oral Rehabil. 2020;47(3):305–12.

[6] Manfredini D, et al. Splint therapy outcomes: limits of evidence. J Oral Facial Pain Headache. 2021;35(2):123–30. 10.11607/ofph.2871 34129656

[7] Ohrbach R, et al. DC/TMD Axis I and II reliability. J Oral Rehabil. 2020;47(2):109–19.

[8] Kerstein RB. Combining digital occlusal analysis and EMG in TMD management. Cranio. 2021;39(1):1–10.33357140

[9] Maga W, et al. Clinical value of digital occlusal analysis in TMD. Diagnostics. 2024;14(3):412.38396451

[10] Shopova D, et al. Digital occlusion tracking in splint therapy. Sensors. 2022;22(18):6912.36146268

[11] Orzeszek S, et al. Efficiency of occlusal splint therapy on orofacial muscle pain. BMC Oral Health. 2023;23:180. 36978070 10.1186/s12903-023-02897-0 PMC10053140

[12] Pficer JK, et al. Occlusal stabilization splints in TMD: long-term outcomes. PLoS ONE. 2017;12:e0171296. 28166255 10.1371/journal.pone.0171296 PMC5293221

